# Acceptability and Feasibility of a Guided Biopsychosocial Online Intervention for Cancer Patients Undergoing Chemotherapy

**DOI:** 10.1007/s13187-020-01792-4

**Published:** 2020-06-18

**Authors:** Miriam Grapp, Friederike Rosenberger, Elena Hemlein, Eva Klein, Hans-Christoph Friederich, Imad Maatouk

**Affiliations:** 1 Department of General Internal and Psychosomatic Medicine, University Hospital Heidelberg, Im Neuenheimer Feld 410, 69120 Heidelberg, Germany; 2 Psycho-oncology Service, National Center for Tumor Diseases (NCT), University Hospital Heidelberg, Im Neuenheimer Feld 460, 69120 Heidelberg, Germany; 3 Working Group Exercise Oncology, Department of Medical Oncology, National Center for Tumor Diseases (NCT), Im Neuenheimer Feld 460, 69120 Heidelberg, Germany; 4 Social Service, National Center for Tumor Diseases (NCT), University Hospital Heidelberg, Im Neuenheimer Feld 460, 69120 Heidelberg, Germany; 5 Nursing Service, National Center for Tumor Diseases (NCT), University Hospital Heidelberg, Im Neuenheimer Feld 460, 69120 Heidelberg, Germany

**Keywords:** Chemotherapy, Feasibility, Guided intervention, Online intervention, Supportive care needs

## Abstract

Chemotherapy is a physically and psychologically highly demanding treatment, and specific Internet-based interventions for cancer patients addressing both physical side effects and emotional distress during chemotherapy are scarce. This study examined the feasibility and acceptability of a guided biopsychosocial online intervention for cancer patients undergoing chemotherapy (OPaCT). A pre-post, within-participant comparison, mixed-methods research design was followed. Patients starting chemotherapy at the outpatient clinic of the National Center for Tumor Diseases in Heidelberg, Germany, were enrolled. Feasibility and acceptability were evaluated through intervention uptake, attrition, adherence and participant satisfaction. As secondary outcomes, PHQ-9, GAD-7, SCNS-SF34-G and CBI-B-D were administered. A total of *N* = 46 patients participated in the study (female 76.1%). The age of participants ranged from 29 to 70 years (*M* = 49.3, SD = 11.3). The most prevalent tumour diseases were breast (45.7%), pancreatic (19.6%), ovarian (13.1%) and prostate cancer (10.8%). A total of *N* = 37 patients (80.4%) completed the OPaCT intervention. Qualitative and quantitative data showed a high degree of participant satisfaction. Significant improvements in the SCNS-SF34 subscale ‘psychological needs’ were found. Study results demonstrate the feasibility and acceptability of the intervention. The results show that OPaCT can be implemented well, both in the treatment process and in participants’ everyday lives. Although it is premature to make any determination regarding the efficacy of the intervention tested in this feasibility study, these results suggest that OPaCT has the potential to reduce unmet psychological care needs of patients undergoing chemotherapy.

**Electronic supplementary material:**

The online version of this article (10.1007/s13187-020-01792-4) contains supplementary material, which is available to authorized users.

## Introduction

Despite significant advances in modern medicine and biomedical science, chemotherapy remains a physically and psychologically highly demanding treatment. Research has consistently found that 30 to 40% of people newly diagnosed with cancer experience a marked degree of psychological distress, including clinically significant depressive or anxiety disorders [1, 2]. Irrespective of cancer type, distress often peaks shortly after cancer diagnosis and during the first 12 months after diagnosis, when a range of medical treatments take place [3]. Psycho-oncology interventions have proven effective in reducing distress, anxiety and depression, and increasing quality of life [4]. However, a high proportion of cancer patients with high levels of psychological distress do not engage with psycho-oncological interventions [5, 6]. Reasons for this include geographical distance from providers, physical limitations or reduced mobility, patients’ preference for managing their emotional and psychological difficulties on their own or stigma about seeking psychological support [7].

A growing body of studies concerning eHealth applications and Internet-based interventions (IBIs) have appeared over the last decade, aimed at improving access to psychosocial support for cancer patients [8, 9]. IBIs in psycho-oncological care vary widely regarding target group and thematic focus, with IBIs focusing on cancer survivors [10], patients with a specific tumour type [11–14], or on specific issues such as sexual functioning [15], insomnia [16] or fatigue [17]. Recently developed eHealth applications and IBIs for patients undergoing chemotherapy have yielded promising results with regard to dealing with chemotherapy-related symptoms (nausea, vomiting, fatigue, hand-foot syndrome etc.) [18–21] or stress management [22].

Although recent studies indicate that cancer patients have multifarious unmet supportive care needs that occur particularly during the neoadjuvant or adjuvant treatment phase [23, 24], this aspect is only marginally addressed in these interventions. Highest demand for support was observed in psychological needs followed by physical and daily living needs as well as health system and information needs [25]. Therefore, we developed a guided biopsychosocial online intervention for cancer patients undergoing chemotherapy (OPaCT). OPaCT is based on the ‘Supportive Care Framework for Cancer Care’ [26]. The intervention is intended to provide orientation and support in dealing with both physical strains and emotional distress, and is aimed at reducing unmet supportive care needs of patients undergoing chemotherapy. The aim of the present study was to investigate the feasibility and acceptability of the OPaCT intervention.

## Methods

### Study Design

We used a pre-post, within-participant comparison, mixed-methods design with pre- and post-treatment questionnaires and semi-structured interviews. The intervention ran from November 2017 to October 2018. The study was conducted in accordance with the Declaration of Helsinki, and the Ethics Committee of the University of Heidelberg (S-320/2017) granted ethical approval. The study was registered at the German Clinical Trials Register (register number: DRKS00013237).

### Participants

German-speaking cancer patients aged 18 years or older, and who started their chemotherapy in the outpatient clinic of the National Center for Tumor Diseases (NCT) in Heidelberg, Germany, were eligible to participate in this study. Participation was independent of stage and severity of tumour disease. Cancer patients with severe psychiatric or cognitive disorders were excluded.

### Recruitment and Procedures

From November 2017 to January 2018, all patients who started chemotherapy at the NCT were informed of the study via flyers, posters and the NCT website. Patients who met the inclusion criteria and gave their written informed consent were included. From February 2018 to July 2018, eligible NCT patients were personally approached by study team members. Patients who agreed to participate in the study were informed about the study goals and procedures and subsequently provided their written informed consent. Reasons for non-participation were documented. After completing a pre-treatment questionnaire (*T*_0_), patients received an email with access to the OPaCT programme. Upon completion of the intervention (or dropping out), a post-treatment questionnaire as well as a semi-structured interview was conducted (*T*_1_).

### Intervention

**Fig. 1:**
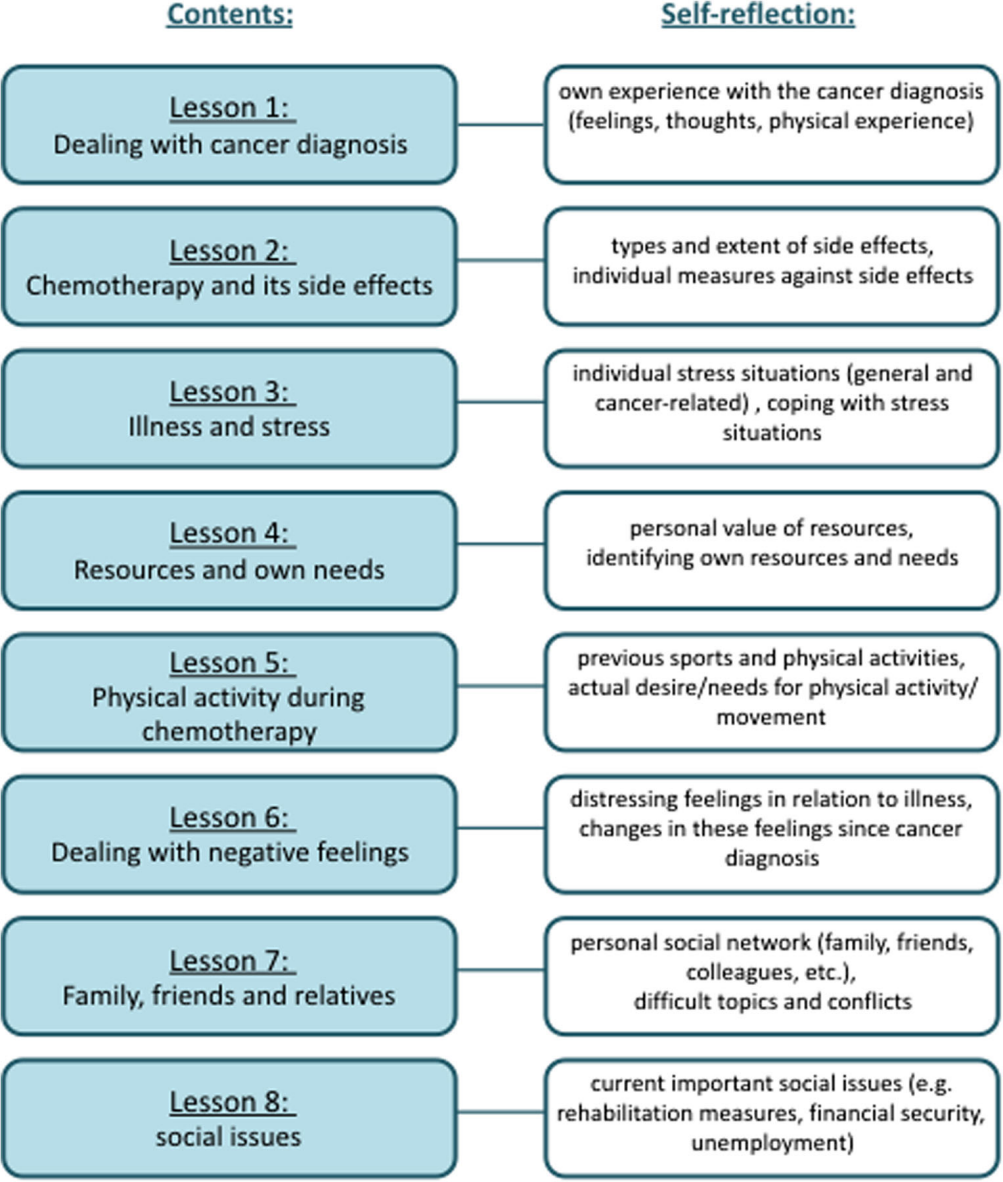
Overview of the OPaCT intervention

OPaCT is built on the theoretical concept of supportive care needs [26]. To cover the broad range of issues relevant to patients undergoing chemotherapy, OPaCT has been co-developed by psychologists, physicians, nurses, social workers and sports scientists. OPaCT comprises psycho-educative as well as interactive supporting elements, daily exercises such as mindfulness and guided imagery exercises or diaries and free-text fields for patients to write down their experiences and feelings (self-reflections). Some illustrations of the visual design of OPaCT and layout-exemplary screenshots are provided in Online Resource 1. The programme is designed as an easily accessible, module-based intervention consisting of eight modules which have to be worked through sequentially. Modules focus on physical and psychosocial issues during chemotherapy (Fig. 1) and are designed to take approximately an hour to complete, depending on level of engagement. A psycho-oncologist, trained in the therapeutic use of electronic media, provided supportive guidance and individualized feedback after completion of each module. Individualized feedback to the patients was provided within 1 to max. 5 days after completion of the module. Feedback referred directly to what the patients wrote in the respective module. Additionally, patients had the opportunity to contact the psycho-oncologist via the messenger of OPaCT at any time.

### Measures

#### Sample Characteristics

Sociodemographic information (sex and age) and the patients’ clinical characteristics were obtained from the clinic’s patient documentation system, which included cancer diagnosis and metastasized cancer (yes/no).

#### Primary Outcomes: Feasibility and Acceptability

Feasibility and acceptability were evaluated through intervention uptake, attrition and adherence as well as participant satisfaction. *Uptake* was assessed as the proportion of patients who agreed to participate in the intervention. *Attrition* was assessed by the number of patients who dropped out before completing the programme. *Adherence* refers to the extent to which the patients engaged with the web-based intervention and was operationalized as number of modules completed and the extent to which participants used the additional elements of OPaCT. *Participant satisfaction* was assessed at post-intervention (*T*_1_) via an author-generated questionnaire containing four Likert-type scales on the following items: (1) ‘OPaCT was helpful to me’, (2) ‘OPaCT provided me with new information’, (3) ‘OPaCT provided practical motivations for everyday life’ and (4) ‘OPaCT could be well integrated into everyday life’. At *T*_1_, the study researcher conducted semi-structured interviews to understand personal experiences as well as potential difficulties with the programme. The interview took approximately 30 min.

#### Secondary Outcomes

At baseline (*T*_0_) and post-intervention (*T*_1_), the following questionnaires were administered: *Depressive syndromes* and *general anxiety disorder* were assessed with the German versions of the Patient Health Questionnaire-9 (PHQ-9) and Generalized Anxiety Disorder (GAD-7) [27]. *Supportive care needs* were assessed with the German version of the Short-Form Supportive Care Needs Survey Questionnaire (SCNS-SF34-G) [28],which is a 34-item self-report questionnaire measuring patients’ perceived type and magnitude of need for support in five domains: health system and information, psychological, physical and daily living, patient care and support, and sexuality needs. *Self-efficacy for coping with cancer* was measured using the German version of the brief form of the Cancer Behavior Inventory (CBI-B-D) [29], which consists of 14 items that describe coping behaviours in the context of cancer (maintenance of independence, participation in treatment decision, stress management and affect management).

### Data Analysis

Statistical analyses were performed with IBM SPSS (version 25) software. Descriptive statistics were obtained for participant demographic, disease and treatment characteristics as well as uptake, attrition, adherence and satisfaction profiles. Reasons for non-participation and attrition were collected. Group differences between participants versus non-participants and completers versus non-completers were analyzed using *t* tests, respectively Mann-Whitney *U* tests for continuous variables, *χ*^2^ tests of independence and Fisher’s exact test for categorical variables. The sample was not powered to detect significance in the outcome measures; nevertheless, we present non-parametric data in relation to distress (depression and anxiety), supportive care needs and self-efficacy to aid understanding of the potential effect of the intervention within this sample and provide data on which a power calculation for a larger study of efficacy can be based. The Bonferroni-Holm method [30] was used to counteract the problem of multiple comparisons.

## Results

### Uptake

**Fig. 2:**
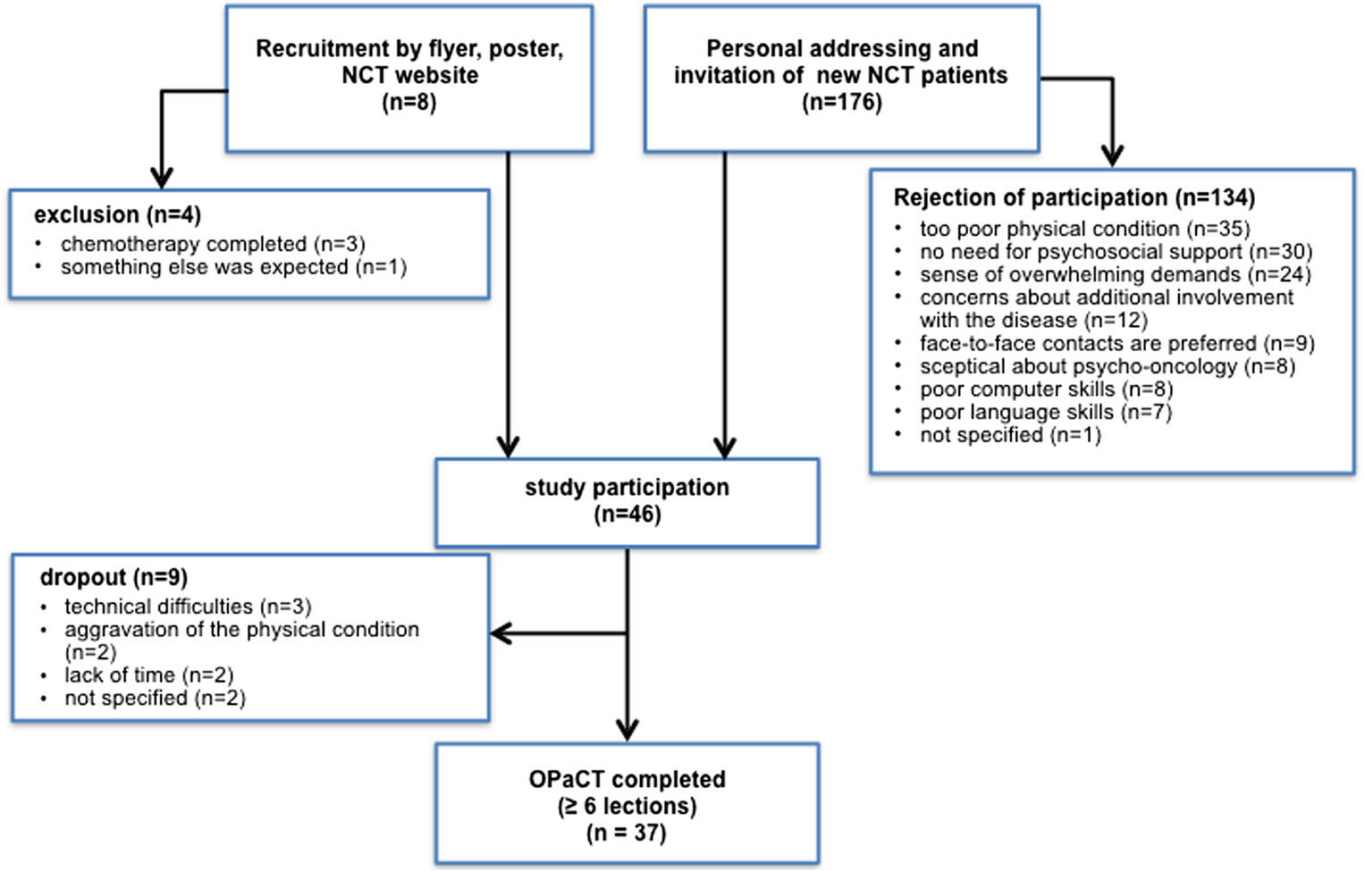
Flow diagram of the uptake and attrition of the OPaCT intervention

From November 2017 to January 2018, eight patients who started chemotherapy at the NCT expressed interest in participating in the study. Of these patients, four (50%) could be included in the study. Reasons for exclusion were already completed chemotherapy (*n* = 3) and divergent expectations of participants (*n* = 1). From February 2018 to July 2018, 176 eligible new NCT patients were personally approached by study team members and informed about the OPaCT study, of whom 42 (23.9%) consented to take part. This resulted in an overall response rate of 25% (46/184). The most common reasons reported for non-participation were too poor physical condition (*n* = 35), no need for psychosocial support (*n* = 30), sense of overwhelming demands (*n* = 24) and concerns about additional involvement with the disease (*n* = 12) (Fig. 2).

### Sample Characteristics

The mean age of participants was 49.2 years (range 29 to 70 years, median 50.5 years). The majority (76.1%) of participants were female. Significant differences in age and gender were found between participants and non-participants: non-participants were significantly older (mean 56.7 years, range 28 to 81 years, median 56.0 years) than participants, and the proportion of women within the non-participants was significantly lower (52.2%). The proportion of patients with metastatic tumour disease was slightly less than one-third among both participants (30.4%) and non-participants (31.9%). There were apparent differences between participants and non-participants with regard to cancer type: the most common tumour diseases among participants were breast (45.7%), pancreatic (19.6%), ovarian (13.1%) and prostate cancer (10.87%). The most common tumour diseases among non-participants were breast (34.1%), stomach/oesophagus (12.7%), skin (8.7%), and head and neck cancer (8.2%) (Table 1).

**Table 1:** Demographic and disease characteristics of participants and non-participants

		Total (*n* = 184)	Participants (*n* = 46)	Non-participants (*n* = 138)	*p*
Age in years (Mean, SD)		54.77 (12.88)	49.24 (11.35)	56.66 (12.87)	0.001
Sex (*n*, %)	Male	77 (41.85%)	11 (23.91%)	66 (47.83%)	0.005
	Female	107 (58.15%)	35 (76.09%)	72 (52.17%)	
Cancer type (*n*, %)	Breast	68 (36.96%)	21 (45.65%)	47 (34.06%)	N/A
	Female genitalia	16 (8.70%)	6 (13.04%)	10 (7.25%)	
	Male genitalia	11 (5.98%)	5 (10.87%)	6 (4.35%)	
	Pancreas	18 (9.79%)	9 (19.57%)	9 (6.52%)	
	Stomach/oesophagus	17 (9.24%)	---	17 (12.32%)	
	Colon/rectum	14 (7.61%)	5 (10.87%)	9 (6.52%)	
	Skin	12 (6.52%)	---	12 (8.70%)	
	Head and neck	11 (5.98%)	---	11 (7.97%)	
	Others	17 (9.24%)	---	17 (12.32%)	
Metastasised (*n*, %)	Yes	58 (31.52%)	14 (30.43%)	44 (31.88%)	0.856
	No	126 (68.48%)	32 (69.57%)	94 (68.12%)	

*p* values from *χ*^2^ and independent samples *t* test

### Attrition

Of the 46 patients registered for the intervention, three (6.6%) never started. Another six (13.3%) completed only one to three lessons, and 37 (80.4%) carried out a substantial part of the intervention (six to eight lessons) (Fig. 2). Reasons reported for dropping out were technical difficulties (*n* = 3), aggravation of the physical condition (*n* = 2), lack of time (*n* = 2) and not specified (*n* = 2). We compared demographic and disease characteristics as well as the baseline scores of the 37 patients who completed at least six of eight lessons of the intervention (‘completers’) with the nine patients who did not complete the intervention (‘non-completers’). No differences were found within sex, age and disease-related variables (time since diagnosis and metastasised condition). There were no differences between completers and non-completers with regard to GAD-7, SCNS-SF34-G and CBI-B-D baseline scores. Only depressive symptoms at *T*_0_ were significantly higher for completers than for non-completers (PHQ-9: *z* = − 2.197, *p* = 0.027).

### Adherence

Over 80% of participants completed a large part of the intervention (six out of eight lessons). Two-thirds (67.39%) of participants carried out the full intervention with all eight consecutive lessons. The majority of participants used the OPaCT element self-reflection (86.96%) and the relaxation exercises (82.61%). About two-thirds of participants (65.22%) regularly communicated via the integrated email function, whereas only a small proportion used the two diaries (34.78% and 26.09%, respectively).

### Participant Satisfaction

**Fig. 3:**
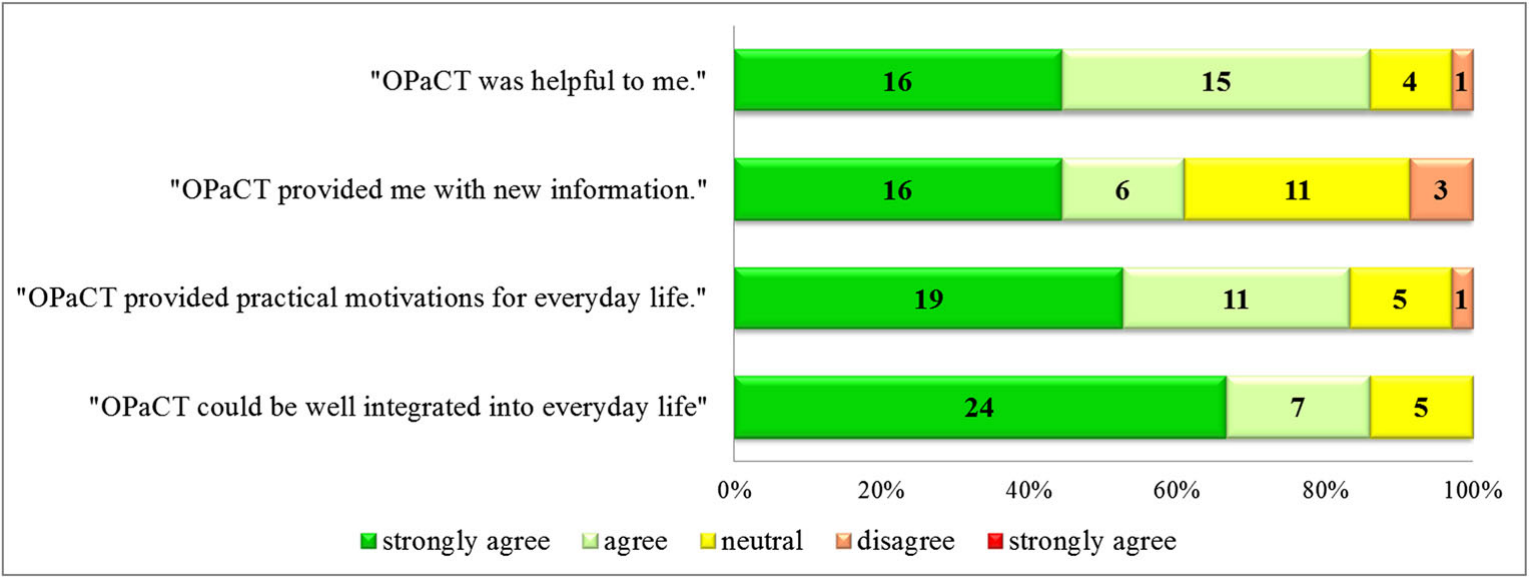
Participant satisfaction with OPaCT intervention

The post-treatment questionnaire was returned by 36 participants (33 completers and three non-completers). Questionnaire responses indicated the majority of patients experienced OPaCT as helpful (86.11%). Most patients found that OPaCT provided practical motivations for everyday life (83.33%) and that it could be well integrated into everyday life (86.11%). Fewer, but still the majority of patients, agreed that OPaCT provided them with new information (61.11%) (Fig. 3).

Additionally, 34 participants (32 completers and two non-completers) agreed to take part in the semi-structured interview at *T*_1_. In the interviews, participants mentioned the following advantages of the web-based intervention: it could be administered flexibly in terms of time and place; it encouraged patients to engage with emotionally difficult issues and, simultaneously, it helped increase awareness of one’s own strengths and resources. The feedback was perceived as highly personalized, and participants felt well understood. According to participants, the intervention could be improved by providing more in-depth information on specific topics, such as nutrition, fatigue, complementary medicine and on dealing with the palliative condition. Some participants missed the possibility of getting into contact with other patients, e.g. via chat, as well as an option of downloading and reading the individual lessons offline. It was also reported that due to fatigue or poor physical condition, the intervention was temporarily experienced as an additional strain, e.g. using the diaries was too time-consuming.

### Secondary Outcomes

Based on 36 returned post-intervention questionnaires, a per-protocol analysis was conducted. Following correction with the Bonferroni-Holm method, the Wilcoxon signed-rank test indicated no improvement in depressive syndromes (PHQ-9: *z* = − 1.614, *p* = 0.800), general anxiety disorder (GAD-7: *z* = − 0.166, *p* = 1.00), overall supportive care needs (SCNS-SF34-G: *z* = − 0.31, *p* = 0.804) and self-efficacy for coping with cancer (CBI-B-D: *z* = − 1.147 *p* = 1.00) at the end of the intervention. Significant improvements from baseline (*T*_0_) to post-intervention (*T*_1_) were found in the SCNS-SF34 subscale ‘psychological needs’ (*z* = − 2.862, *p* = 0.036) (see Table 2).

**Table 2:** PHQ-9, GAD-7, SCNS-SF34 and CBI-B-D scores baseline (*T*_0_) and post-intervention (*T*_1_)

	*T*_0_			*T*_1_			
	Med	Mean	SD	Med	Mean	SD	*p* value
PHQ-9 score	6.00	6.46.	3.73	6.00	6.08	3.83	.800
GAD-7 score	5.00	5.28	3.67	4.00	5.29	3.79	1.00
SCNS-SF34 score	71.00	75.89	26.45	69.00	66.52	17.75	.804
SCNS-SF34 subscales:							
Health system and information needs	20.00	24.16	11.23	20.00	21.51	9.11	1.00
Psychological needs	24.00	26.60	10.55	19.50	20.59	8.06	.036
Physical and daily living needs	9.00	9.72	3.92	9.00	9.46	2.66	1.00
Patient care and support needs	7.50	8.78	4.24	7.00	7.42	2.71	.800
Sexuality needs	5.00	6.54	3.86	5.00	5.75	2.71	1.00
CBI-B-D score	91.50	93.30	20.24	93.30	91.03	19.92	1.00

*p* values from Wilcoxon signed-rank test with Bonferroni-Holm correction.; *Med*, median; *PHQ-9*, depression module of the Patient-Health Questionnaire; *GAD-7*, anxiety module of the Patient Health Questionnaire; *SCNS-SF34-G*, German version of the Short-Form Supportive Care Needs Survey Questionnaire; *CBI-B-D*, German version of the brief form of the Cancer Behavior Inventory

## Discussion

In the present study, we investigated the feasibility and acceptability of a guided biopsychosocial online intervention for cancer patients undergoing chemotherapy (OPaCT). The results of this feasibility study reveal initially a low uptake with a response rate of 25% but subsequently a high adherence and a completer rate of over 80%. The post-treatment questionnaire indicated a high level of patient satisfaction with the intervention. The intervention was experienced as helpful, and it could be well integrated into everyday life. Participants showed significant improvement with regard to unmet psychological needs. The low uptake of OPaCT is in line with findings from other studies of IBIs. Ebert et al. [31] indicated that the uptake rates of IBIs for depression are currently rather low, varying between 3 and 25%. Recent studies in cancer patients also encountered great difficulties regarding recruitment and a low participation rate in IBIs [14, 32]. In addition to the low uptake, two common reasons for non-participation were ‘sense of overwhelming demands’ and ‘concerns about additional involvement with the disease’. This raises the question of the optimal time frame for intervention: should OPaCT occur as early as possible in order to optimally support patients during chemotherapy, or would it be more reasonable to offer the intervention in the course of chemotherapy if a certain ‘routine’ with the treatment has been established? The low dropout rate of 19.6% and the high adherence rate of 80.4% in our study are noteworthy, especially because high attrition and low adherence are considered a main limitation of online self-help interventions in both cancer and non-cancer populations [33]. There is some evidence that an increased level of guidance or support leads to better adherence, and some studies emphasize the superiority of guided interventions over unguided interventions [34]. A core element of OPaCT is personal feedback, which the patients experienced as particularly supportive and beneficial. We therefore assume that the intensive and highly individualized personal guidance provided for the patients led to the high adherence and the low dropout rate.

About half of our participants were breast cancer patients, while patients with skin, stomach/oesophagus, or head and neck cancer were not represented. These results are consistent with findings that patient characteristics such as sex, age and tumour site are linked to the uptake of psycho-oncological support [35–37]. In our study sample, 15.2% of participants had a clinical indication for anxiety (GAD-7) and 21.8% for depression (PHQ-9). This is a lower percentage of highly distressed patients than in other studies reporting psychological distress of people newly diagnosed with cancer [1, 2], but comparable with other studies on online-based interventions [10, 38]. Comparing baseline scores of the completers and non-completers in our sample, however, we found that the PHQ-9 score at the beginning of the intervention was significantly higher for completers than non-completers. These results suggest that highly distressed patients, although they make less extensive use of OPaCT, may particularly benefit from the intervention.

In recent years, a small number of IBIs were developed for patients undergoing chemotherapy, most of them focusing on management of chemotherapy-related symptoms or stress management [39]. The distinctive aspect of OPaCT is that the intervention is based on the Supportive Care Framework for Cancer Care [26] and focuses both on the physical side effects of chemotherapy and on emotional distress during treatment. The main strength of our study is that it is a biopsychosocial intervention developed on an interdisciplinary basis which addresses a broad group of patients. The intervention is patient oriented and can be used in clinical care independently of research. Another strength of this study is that we used a mixed-methods research design and collected multifaceted data regarding the feasibility and the acceptability of OPaCT. This enabled a good insight into which patients participated in the intervention and what the participating patients experienced as particularly helpful.

Some limitations of the study need to be acknowledged. Because feasibility and acceptability of the intervention were the main focus of our study, a single-arm study was conducted. There was no control group, and the sample size was small and not powered to detect changes in secondary outcome criteria. Although not essential for many aspects of this study, the inclusion of a control group would have allowed a more realistic examination of recruitment, randomization and implementation of the intervention. Our study sample in this pilot study was wide ranging: no specification was made for certain tumour types or for particularly distressed patients. The intervention should initially be made available to a large group of patients whose common denominator is the beginning of chemotherapy with its possible physical and psychological burdens. We accepted the resulting heterogeneity of our study sample to (1) prevent overlooking a small but important group of patients who might benefit from the intervention and (2) investigate whether there are specific patient groups (e.g. depending on cancer type) who use OPaCT in particular.

## Conclusion

In summary, the guided biopsychosocial online intervention for cancer patients undergoing chemotherapy (OPaCT) has proven feasible and our results indicate that OPaCT can be implemented well both in the treatment process and in patients’ everyday lives. The intervention has the potential to provide orientation and support in dealing with physical strains and emotional distress, and is aimed at reducing unmet supportive care needs of patients undergoing chemotherapy. The clinical effectiveness of OPaCT needs to be tested in an evaluative research design. This study showed that recruitment was more difficult than expected and that recruitment procedures should be carefully considered when planning a randomized controlled trial.

## Electronic Supplementary Material

ESM 1 (PDF 889 kb)

ESM 2 (XLSX 12 kb)

## Data Availability

The authors have full control of all primary data and agree to allow the journal to review all data if requested.
