# Supplementary material for: Acceptability and Feasibility of a Guided Biopsychosocial Online Intervention for Cancer Patients Undergoing Chemotherapy
Source: J Cancer Educ. 2020 Jun 18;37(1):102–10. doi: 10.1007/s13187-020-01792-4 (PMC8816767; doi:10.1007/s13187-020-01792-4)
Supplement: Supplementary file 1 — (PDF 889 kb) [file 13187_2020_1792_MOESM1_ESM.pdf]

OPaCT intervention design and layout-exemplary screenshots

Note: The OPaCT was developed in German and the screenshots are, therefore, in German. However, please find the translation of key points alongside the example screenshots below:

Screenshot 1: OPaCT introduction and general overview page

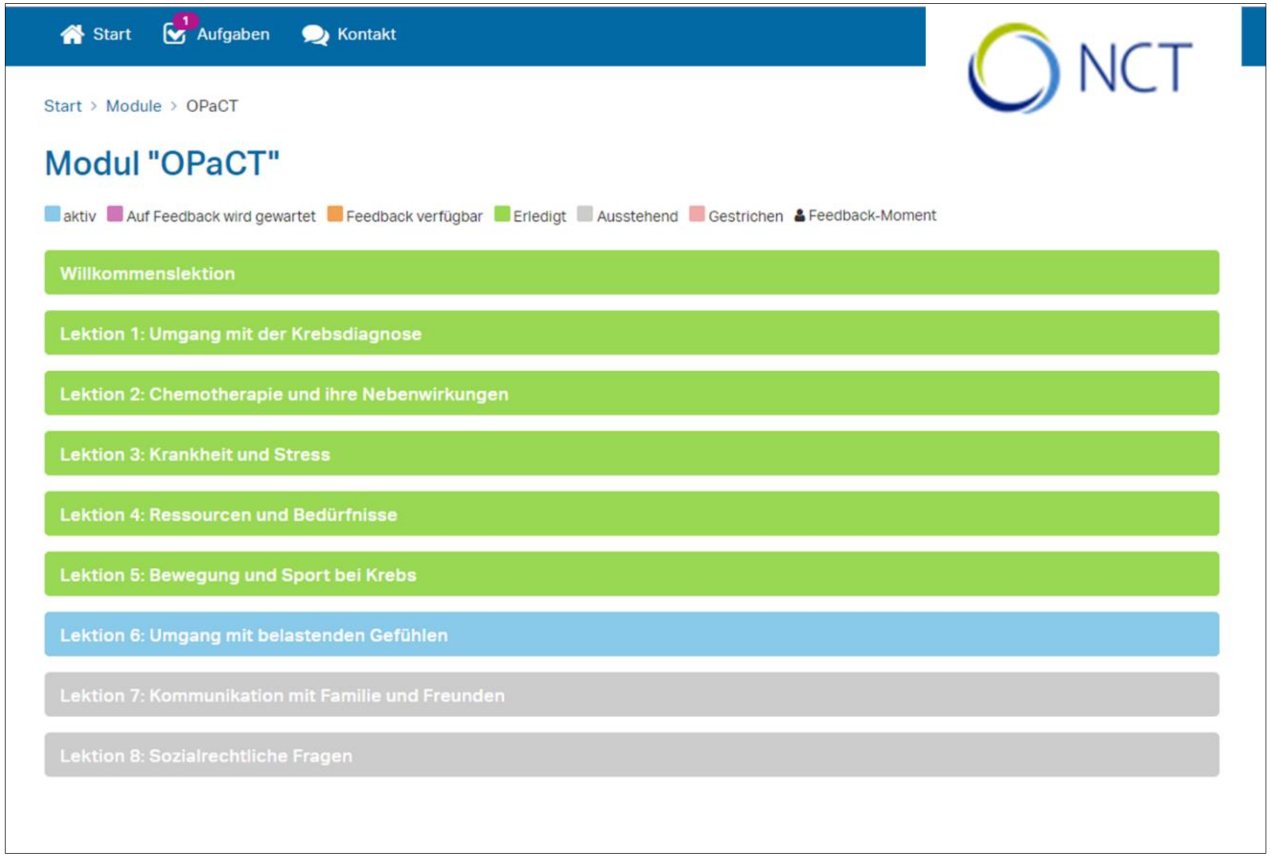

Module „OPaCT“

Captions: active *(blue)* - waiting for feedback *(pink)* - feedback available *(orange)* - completed *(green)* - pending *(gray)* - deleted *(red)* - feedback moment

Introductory lesson

Lesson 1: Dealing with cancer diagnosis

Lesson 2: Chemotherapy and its side effects

Lesson 3: Illness and stress

Lesson 4: Ressources and own needs

Lesson 5: Physical activity during chemotherapy

Lesson 6: Dealing with negative feelings

Lesson 7: Communication with family members and friends

Lesson 8: Social issues

Screenshot 2: Psycho-educational elements (“Illness and stress”)

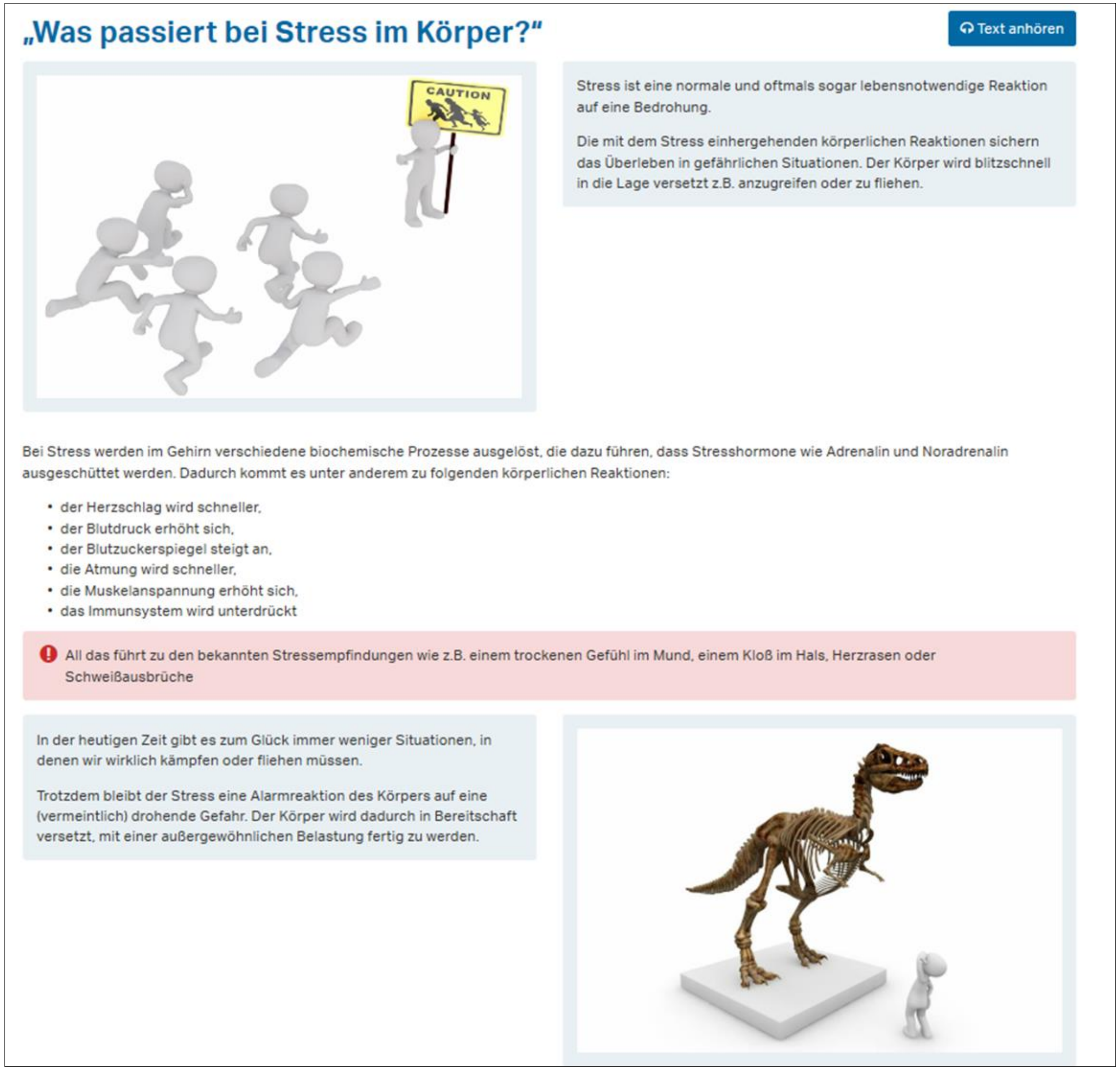
„Was passiert bei Stress im Körper?“

Text anhören

Stress ist eine normale und oftmals sogar lebensnotwendige Reaktion auf eine Bedrohung.

Die mit dem Stress einhergehenden körperlichen Reaktionen sichern das Überleben in gefährlichen Situationen. Der Körper wird blitzschnell in die Lage versetzt z.B. anzugreifen oder zu fliehen.

Bei Stress werden im Gehirn verschiedene biochemische Prozesse ausgelöst, die dazu führen, dass Stresshormone wie Adrenalin und Noradrenalin ausgeschüttet werden. Dadurch kommt es unter anderem zu folgenden körperlichen Reaktionen:

- der Herzschlag wird schneller,
- der Blutdruck erhöht sich,
- der Blutzuckerspiegel steigt an,
- die Atmung wird schneller,
- die Muskelanspannung erhöht sich,
- das Immunsystem wird unterdrückt

! All das führt zu den bekannten Stressempfindungen wie z.B. einem trockenen Gefühl im Mund, einem Kloß im Hals, Herzrasen oder Schweißausbrüche

In der heutigen Zeit gibt es zum Glück immer weniger Situationen, in denen wir wirklich kämpfen oder fliehen müssen.

Trotzdem bleibt der Stress eine Alarmreaktion des Körpers auf eine (vermeintlich) drohende Gefahr. Der Körper wird dadurch in Bereitschaft versetzt, mit einer außergewöhnlichen Belastung fertig zu werden.

How does stress affect the body?

Stress is a normal and sometimes life-saving response to danger.

The physical reactions associated with stress help ensure survival in dangerous situations. Our body is immediately able to attack or flee.

Stress triggers various biochemical processes in the brain which result in the release of stress hormones, such as adrenaline and noradrenaline. This leads to the following physical reactions:

- heartbeat increases,
- blood pressure increases,
- blood sugar level rises,
- breathing becomes faster,
- muscle tone increases,
- immune system is suppressed

! This leads to familiar stress symptoms including feeling like your mouth is dry, you have a lump in your throat, heart palpitations or sweating.

Luckily these days there are fewer and fewer situations where we really have to fight or flee.However, stress is the body's warning signal in response to an (ostensibly) imminent danger. As a result, the body is equipped to cope with extraordinarily stressful situations.

Screenshot 3: free-text fields for patients to write down their experiences and feelings (self-reflections) (lesson 6 “Dealing with negative feelings”)

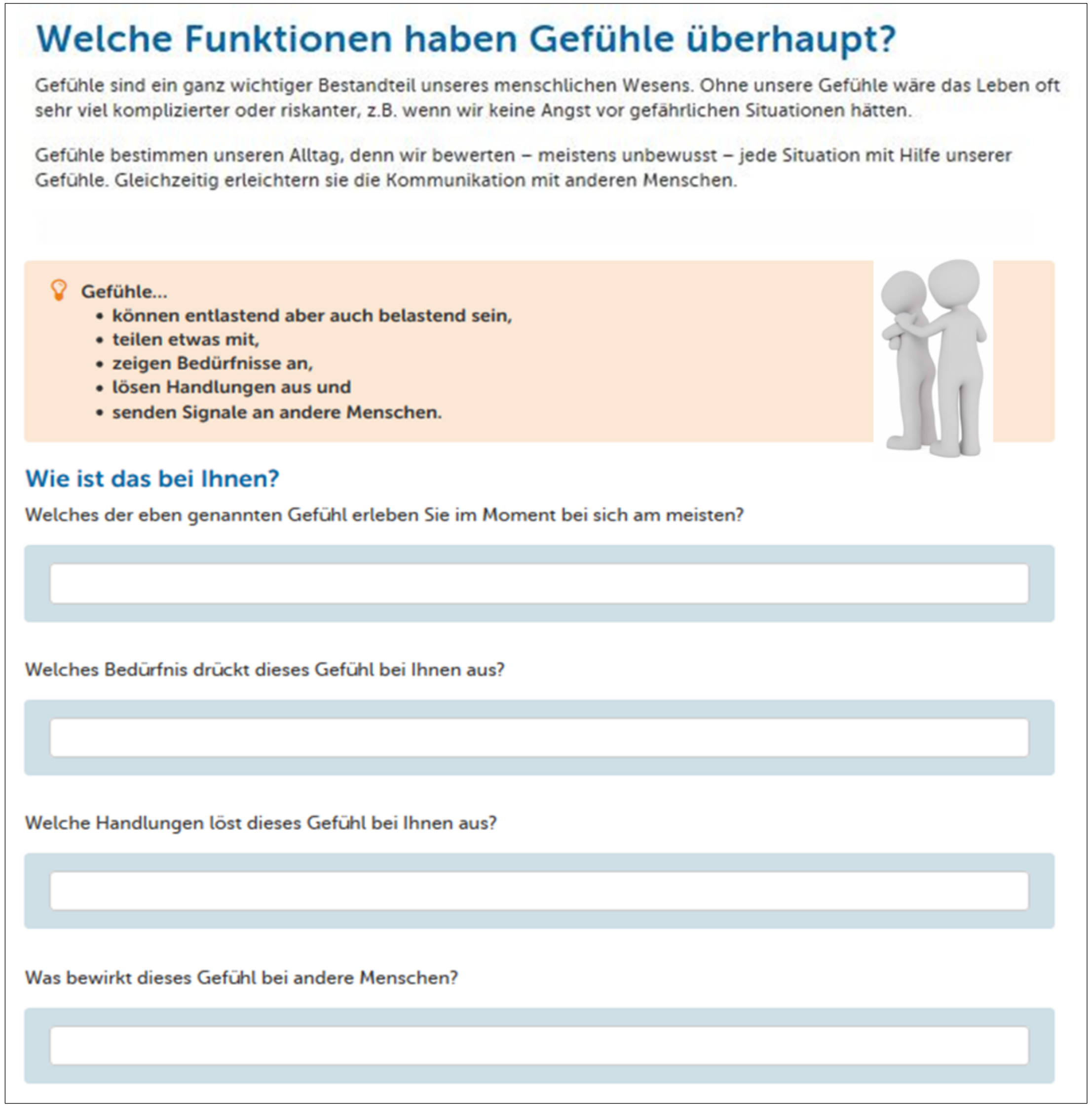

Welche Funktionen haben Gefühle überhaupt?

Gefühle sind ein ganz wichtiger Bestandteil unseres menschlichen Wesens. Ohne unsere Gefühle wäre das Leben oft sehr viel komplizierter oder riskanter, z.B. wenn wir keine Angst vor gefährlichen Situationen hätten.

Gefühle bestimmen unseren Alltag, denn wir bewerten – meistens unbewusst – jede Situation mit Hilfe unserer Gefühle. Gleichzeitig erleichtern sie die Kommunikation mit anderen Menschen.

Gefühle...

- **können entlastend aber auch belastend sein,**
- **teilen etwas mit,**
- **zeigen Bedürfnisse an,**
- **lösen Handlungen aus und**
- **senden Signale an andere Menschen.**

Wie ist das bei Ihnen?

Welches der eben genannten Gefühl erleben Sie im Moment bei sich am meisten?

Welches Bedürfnis drückt dieses Gefühl bei Ihnen aus?

Welche Handlungen löst dieses Gefühl bei Ihnen aus?

Was bewirkt dieses Gefühl bei andere Menschen?

So what are emotions good for exactly?

Emotions are an important part of our human nature. Without our emotions, life would be much more complicated and dangerous. Just think what could happen if we were no longer frightened by dangerous situations.

Emotions shape our everyday lives because we - usually unconsciously assess - every situation according to our current feelings.

Emotions...

- can be both a relief and a burden,
- can communicate something,
- specify needs,
- initiate actions and
- send signals to others.

What about you?

Which of the feelings mentioned above are you experiencing the most at the moment?

With which need is this feeling associated for you?

What kind of action does this feeling initiate in you?

What could this feeling cause in others?

Screenshot 4: interactive supporting elements (lesson 6 “Dealing with negative feelings”)

Aufgabe: Seelischer Notfallkoffer

Hilfreich im Umgang mit belastenden Gefühlen kann ein ganz persönlicher „seelischer Notfallkoffer" sein, auf den Sie in belastenden Situationen zurückgreifen können.

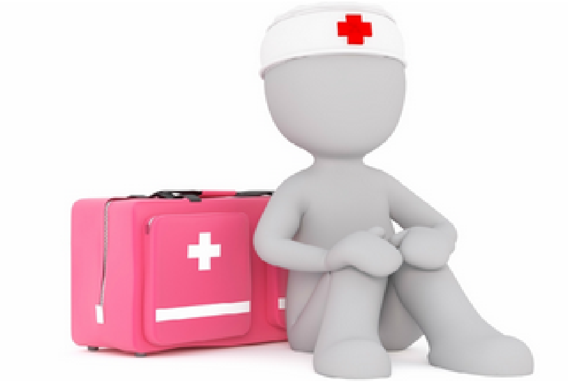

Der seelische Notfallkoffern beinhaltet diejenigen Dinge, die Ihnen in guttun, wenn es Ihnen schlecht geht. In diesen Koffer können SIe auch Dinge tun, die Ihnen früher (z.B. als Kind oder Jugendlicher) in schwierigen Situationen gutgetan haben.

Folgendes könnte in einen solchen seelischen Notfallkoffer hinein kommen:

- Bilder von Landschaften, die Sie mögen,
- Musik, die Sie gerne hören,
- Düfte, die Sie gerne riechen,
- Dinge, die Sie gerne berühren (z.B. Steine, Kastanien, Muscheln, Kissen, Stofftiere,...),
- Dinge, die Sie gerne essen oder trinken,
- ein Kleidungsstück, in dem Sie sich wohlfühlen,
- Bilder oder Briefe von lieben Menschen,
- alles, was Ihnen sonst noch einfällt.

Nehmen Sie sich einen Moment Zeit und notieren Sie hier, was in Ihren seelischen Notfallkoffer hineinkommen soll:

Exercise: Your emotional emergency kit

A personal "emotional emergency kit" can be extremely helpful when coping with stressful feelings.

An emotional emergency kit is packed with all the things that might help you when you are feeling low or stressed. Your emotional emergency kit may also contain things that have helped you through difficult situations in the past (e.g. in childhood or adolescence).

Here are some of the things that you might want to include in your emotional emergency kit:

- - Pictures of landscapes that you like,
- - Music you enjoy listening to,
- - Fragrances you like to smell.
- - Things you like to touch (e.g. stones, chestnuts, shells, pillows, soft toys, ...)
- - Things you like to eat or drink,
- - A piece of clothing in which you feel comfortable
- - Pictures and letters from loved ones,
- - Anything else you can think of

Now take a moment and write down what you intend to put into your emotional emergency kit.

Screenshot 5: Mindfulness and guided imagery exercises (Lesson 3 “Illness and stress”)

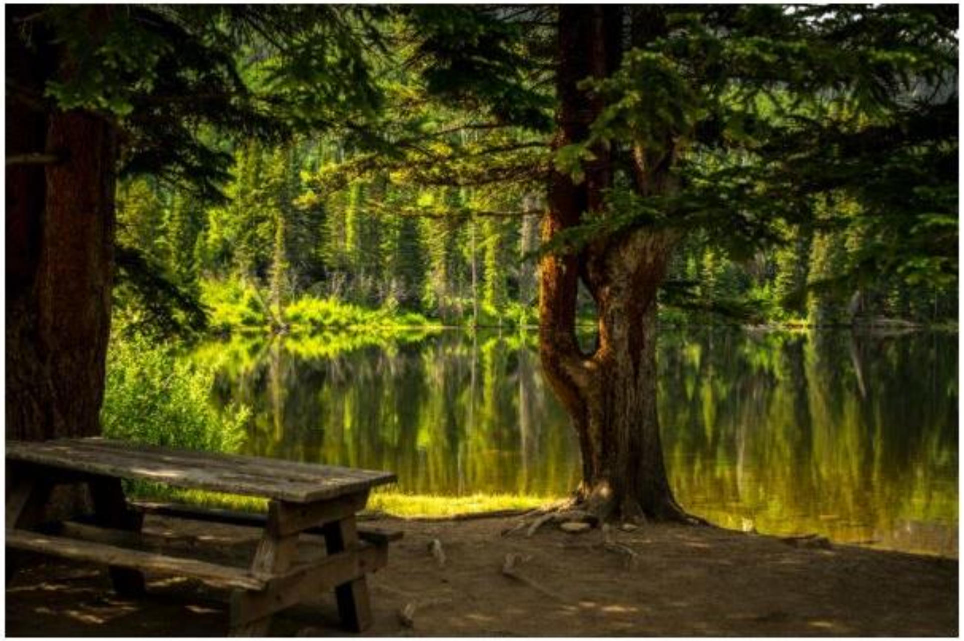

Auch die dritte Lektion haben Sie nun erfolgreich bearbeitet. Sie haben darin einiges über Stress und seine Auswirkungen auf unseren Körper, unser Denken und unser Fühlen erfahren.

Für viele Menschen sind bei Stress Entspannungs- und Imaginationsübungen hilfreich. Daher möchten wir Ihnen ab jetzt am Ende jeder Lektion eine Entspannungsübung anbieten. Die Entspannungsübung in dieser Lektion heißt "Der sichere Ort". Sie können jederzeit in diese Lektion zurückkommen und diese Entspannungsübungen wiederholen, wenn sie Ihnen gut getan hat.

Hier starten Sie den Entspannungsübung "Der sichere Ort".

-7:52

Now you have completed the third lesson. You have learned a lot about stress and its effects on our body, our thinking and our feelings.

When under stress, many people experience mindfulness and guided imagery exercises as helpful and relaxing. From now on, we would therefore like to offer you a mindfulness exercise at the end of every lesson. In this lesson, the exercise is called "the inner safe place" and uses guided imagery. Feel free to come back to this lesson and repeat the exercise at any time if you feel comfortable with it.
